# Denosumab for management of severe hypercalcemia in primary hyperparathyroidism

**DOI:** 10.1530/EC-20-0380

**Published:** 2020-10-01

**Authors:** Anna Eremkina, Julia Krupinova, Ekaterina Dobreva, Anna Gorbacheva, Ekaterina Bibik, Margarita Samsonova, Alina Ajnetdinova, Natalya Mokrysheva

**Affiliations:** 1 Endocrinology Research Center, Russian Federation, Moscow, Russia; 2 Faculty of Fundamental Medicine, ederal State Budget Educational Institution of Higher Education M.V. Lomonosov Moscow State University, Moscow, Russia

**Keywords:** denosumab, primary hyperparathyroidism, hypercalcemia, parathyroid cancer

## Abstract

Hypercalcemic crisis is a severe but rare complication of primary hyperparathyroidism (PHPT), and data on denosumab treatment of patients with this disease is still very limited. The aim of this paper is to investigate the hypocalcemic effect of denosumab in PHPT patients with severe hypercalcemia when surgery should be delayed or is impossible for some reasons. We performed a retrospective study of 10 patients. The analysis included the use of biochemical markers of calcium-phosphorus metabolism, which were followed after the administration of 60 mg of denosumab. The trend to calcium reduction was already determined on the 3rd day after denosumab administration. In most cases the decrease in serum calcium level to the range of 2.8 mmol/L on average or lower was observed on the 7th day (*P* = 0.002). In addition to a significant increase in calcium levels we confirmed a significant increase in the estimated glomerular filtration rate on 7th day (*P* = 0.012). After that, seven patients underwent successful parathyroidectomy and achieved eucalcemia or hypocalcemia, one patient developed the recurrence of parathyroid cancer after initial surgery, while two patients with severe cardiovascular pathology refused surgery. Our study shows that denosumab is a useful tool in PHPT-associated hypercalcemia before surgery or if surgery is contraindicated.

## Introduction

Primary hyperparathyroidism (PHPT) and malignancy are the two most common causes of hypercalcemia (1). Hypercalcemic crisis (HC) is an uncommon, but severe complication of PHPT more specifically for parathyroid cancer (2). The risks of HC are significantly increased in patients with total albumin-corrected calcium >3.5 mmol/L or ionized calcium >2.5 mmol/L. Hypercalcemia may be associated with a spectrum of clinical manifestations, ranging from minor symptoms in the case of mild chronic hypercalcemia to dramatic symptoms as confusion and lethargy in HC, possibly leading to coma and death. The rate of increase of serum calcium often determines the symptoms and the required therapeutic interventions (3, 4).

Parathyroidectomy is the only cure for severe hypercalcemia, or HC caused by PHPT. Removing the parathyroid tumor significantly lowers the levels of parathyroid hormone (PTH) in blood, and thereby decreases serum calcium (5). However, immediate treatment for severe hypercalcemia or an HC should begin with adequate hydration with isotonic saline. Therapy with hypocalcemic drugs is also an option, which allows the patient to prepare for surgery. Bisphosphonates are widely used and approved for the treatment of hypercalcemia, but they have some limitations such as significant nephrotoxicity, like pamidronate-induced glomerulosclerosis and acute tubular necrosis. Although cinacalcet has been successfully applied in PHPT, it has been discontinued prematurely due to its side effects. Moreover, its hypocalcemic effect of cinacalcet is usually not immediate since step-by-step dose titration is most often required which may take several weeks in some cases, and the dose escalation is hugely limited by nausea side effect (6, 7).

The data on the efficacy and safety of denosumab led to its approval for postmenopausal osteoporosis, skeletal complications and hypercalcemia of malignancies. In case of postmenopausal osteoporosis denosumab reduces the risk of vertebral, non-vertebral and hip fractures and increases bone mineral density (8). At the same time, administering denosumab to patients with advanced cancers and bone metastases significantly decreases the incidence of skeletal-related events and hypercalcemia. It is, occasionally, the only way to lower calcium levels and achieve stable results. Unlike other anti-hypercalcemic drugs, denosumab can be used in the case of renal dysfunction (9).

The aim of this paper is to investigate the hypocalcemic effect of denosumab in PHPT patients with severe hypercalcemia then surgery should be delayed or is impossible for some reasons. Data on denosumab use in patients with PHPT is limited. The RANKL/RANK signaling pathway has a key role in the PHPT pathogenesis because of direct bone resorptive effects of increased RANKL in this disease. Denosumab binds RANKL with high affinity and specificity, preventing interaction with RANK on the osteoclast membrane. By blocking RANKL, denosumab inhibits osteoclast differentiation, activation and survival. This prevents bone resorption and thereby decreases blood calcium levels (10).

## Materials and methods

We performed a retrospective study of all patients who received subcutaneously 60 mg of denosumab (Prolia®) for severe or symptomatic hypercalcemia due to PHPT to analyze the effects of denosumab on calcium levels. Ten patients were identified from the inpatient medical record system. Data were collected from January 2016 to June 2020 at the Department of Parathyroid Pathology.

The analysis included the biochemical markers of calcium-phosphorus metabolism: albumin-adjusted serum calcium concentrations (Ca, reference range (RR) 2.15–2.55 mmol/L), phosphate (P, RR 0.74–1.52 mmol/L), total alkaline phosphatase (AP, RR 40–150 U/I), intact parathyroid hormone (iPTH, RR 15–65 pg/mL), creatinine (RR 63–110 for men and 50–93 for women, mcmol/L), glomerular filtration rate calculation using the CKD-EPI creatinine equation (eGFR, mL/min/1.73 m^2^), and 24-h urinary calcium excretion for patients with eGFR >60 mL/min/1.73 m^2^ (RR 2.5–8 mmol/day). All parameters are shown as a median (minimum, maximum). The albumin-corrected serum calcium was calculated according to the following formula: serum total calcium (mg/dL) − 0.8 × (serum albumin (g/dL) − 4.0). In most cases biochemical parameters were measured at the baseline (Ca, P, AP, creatinine, eGFR, iPTH, 24-h urinary calcium), on the 3rd (Ca) and 7th day after denosumab administration (Сa, creatinine and eGFR). For laboratory tests the biochemical analyzer ARCHITECH c8000 (Abbott) and the electrochemiluminescence analyzer Cobas 6000 (Roche) were used. In four patients, calcium levels were available only on 9th day after injection. Further tests were conducted after surgery (if it was performed) or several months after the denosumab injection (if the surgical treatment was delayed for some reason). Severe hypercalcemia was defined as total albumin-corrected calcium levels >14 mg/dL (3.5 mmol/L). Symptomatic hypercalcemia was defined as an occurrence of hypercalcemic-related symptoms regardless of the achievement of calcium levels >14 mg/dL (3.5 mmol/L). Hypercalcemic-related symptoms included cognitive impairment and irritability, dizziness, nausea and vomiting, severe dehydration, cardiovascular disturbances. The diagnosis of osteoporosis was established based on the results of the radiography (Axiom R200, Siemens) and dual X-ray absorptiometry (Lunar iDXA, GE Healthcare). Bone mineral density (BMD) was measured at the lumbar spine (LS), femoral neck (FN), total hip (TH) and radius 33% (R33%). The X-ray or CT scans with lytic or multilobular cystic changes in bones confirmed the presence of osteitis fibrosa cystica. Baseline patient characteristics also included the low energy fractures, kidney stones and prior therapy of PHPT.

The study was approved by the Ethics Committee of the Endocrinology Research Centre (Moscow, Russia; protocol #1 of 17/01/2018). Informed consent has been obtained from each patient after full explanation of the purpose and nature of all procedures used.

Statistical analysis was performed with the use of Statistica v. 13.3 (TIBCO Software Inc., Palo Alto, CA, USA). Comparison of two dependent groups for quantitative data was carried out using the Wilcoxon criterion, comparison of three dependent groups for quantitative data was carried out using the Friedman criterion. The initial critical level of significance in testing statistical hypotheses was assumed to be 0.05. At multiple comparisons of the parameters, it was recalculated with Bonferroni correction (*P* = 0.017).

## Results

### Clinical characteristics

The median age of the patients was 53.5 years (min 25, max 79), two of them were males and eight females. Seven patients presented with multiple erosions of the stomach or duodenum, as well as moderate and severe anemias, which required correction before surgery and postponed urgent surgical treatment for PHPT. The patients received an injection of denosumab due to the development of life-threatening symptoms to stabilize their condition. In the examined group, one female patient had an atypical adenoma, two patients had morphologically confirmed parathyroid cancer, and five patients had typical adenoma of parathyroid gland. In the two other cases, the morphological diagnosis remains unknown due to refusal of surgery.

In the studied group the baseline median Ca concentration was 3.8 mmol/L (3.4;5.0), the iPTH – 933 pg/mL (345; 2146). The other baseline parameters were as follows: P 0.79 mmol/L (0.31; 1.18), AP 174.5 U/L (69; 514), creatinine 116.8 mcmol/L (34; 296.8), eGFR 45 mL/min/1.73 m^2^ (19;159), 24-h urinary calcium was available in only two patients and amounted to 7.9 and 10.9 mmol/day, respectively. Table 1 summarizes the main laboratory and instrumental results.

**Table 1:** Demographic, biochemical, clinical and histopathological profile in hypercalcemic patients.

Case	Sex	Age^a^	Ca at baseline	Ca on 3rd day^b^	Ca on 7th day^b^	Ca on 9th day^b^	eGFR baseline	eGFR on 7th day^b^	PTH baselina	PTH a.i.	AP	PHPT complications	Hypercalcemic-related symptoms	Morphological diagnosis
1	F	79	3.5	2.85	2.48		36	56	513		69	Osteoporosis (L1–L4 −2.9 s.d., radius 33% −3.4 s.d. in T-score), kidney stones impaired renal function	Cognitive impairment, headaches, nausea and vomiting, dehydration, hypotension	Adenoma
2	M	75	4.42	3.78	2.58		28	48	773	667	155	Impaired renal function	Cognitive impairment, dehydration	Adenoma
3	F	44	3.34	2.46	2.42		44	61	1052		514	Osteoporosis (radius 33% −3.2 s.d. in Z-score), kidney stones impaired renal function	Cognitive impairment, headaches, dizziness, nausea and vomiting, dehydration	Adenoma
4	F	30	3.94	2.94	3		124	132	1423	1480	502	Osteoporosis (L1–L4 −4.9 s .d., radius 33% −4.6 s.d. in Z-score), low energy fractures (both femurs), osteitis fibrosa cystica, kidney stones	Dehydration, irritability, dizziness	Adenoma
5	F	25	3.43	3.23	2.75	2.39	139	144	567	665	495	Osteoporosis (L1–L4 -6.1 s.d., femur neck −4.9 s.d., total hip −5.8 s.d., radius 33% −7.6 s.d. in Z-score), low energy fractures (vertebral Th7-Th10, L4, radius and ulna)	Nausea, irritability, dehydration	Adenoma
6	F	65	3.6	3.19	3		46	58	1871	5000	461	Osteoporosis (L1–L4 −4.3 s .d., total hip −2.8 s.d. in T-score), low energy fractures (humerus, radius, ribs), osteitis fibrosa cystica, kidney stones, impaired renal function	Dehydration, arrhythmia	Atypical adenoma
7	F	40	3.99	3.58	2.75	2.44	39	63	1112	1318	2.66	Osteoporosis (femur neck −3.3 s.d., Total hip −3.9 s.d., radius 33% −4.4 s.d. in Z-score), low energy fractures (femur), osteitis fibrosa cystica, kidney stones, impaired renal function	Nausea, irritability, headaches, dehydration	Adenoma
8	M	61	4.11	3.5	2.82	2.47	19	21	2146	1297	194	Osteoporosis (radius 33% −4.5 s.d., femur neck −3.9 s.d. in T-score), low energy fractures (radius), kidney stones, impaired renal function	Cognitive impairment, headaches, nausea, irritability, dehydration	Parathyroid cancer
9	F	46	3.4		2.73		85		345		82	Osteoporosis (radius 33% −2.4 s.d. in Z-score)	Dehydration, dizziness	Parathyroid cancer (at the admission – recurrence with an unidentified metastases)
10	F	68	5			2.73	57	−72 on 9th day	814	414	145	Osteoporosis (vertebral deformations, low energy fractures (radius)), kidney stones. impaired renal function	Nausea, irritability, dehydration	Adenoma

^a^Age at the admission, ^b^After denosumab injection.

Ca, albumin-adjusted serum calcium, RR 2.15–2.55 mmol/; iPTH, intact parathyroid hormone, RR 15–65 pg/mL; AP, total alkaline phosphatase, RR 40–150 U/L; eGFR, glomerular filtration rate using the CKD-EPI creatinine equation (eGFR, mL/min/1.73 m^2^); a.i., after injection before surgery.

As expected, most patients showed signs of bone disease. A significant BMD decrease in various parts of the skeleton occurred in nine individuals. Six patients had a history of low energy fractures and three of them had the radiographic features of osteitis fibrosa cystica and brown tumors. One young female patient had multiple vertebral fractures, while in two cases there were the initial signs of vertebral compression with a maximal body height loss of 15%. Two patients had pathological fractures of the femur followed by mobility limitation. Other fractures affected clavicula, radius, ulna and ribs (Table 1). Seven patients had kidney stones. Most stones were less than 10 mm in diameter. An eGFR less than 60 mL/min/1.73 m^2^ was observed in 70% (7/10) of patients, three of whom had stage 4 CKD (eGFR 15–29 mL/min/1.73 m^2^). The most frequent hypercalcemic-associated symptoms were dehydration (100%), nausea (60%), vomiting (20%) and irritability (50%). Neurologic symptoms (mild cognitive impairment and headaches as well as dizziness) occurred in 40 and 30%, respectively. One woman presented with arrhythmia (P. No 6) (Table 1).

### Treatment

All patients received a 60 mg single dose of denosumab. They were also treated with isotonic saline (1000–1500 mL/24 h) and cinacalcet in various doses. In half of all cases, a 30 mg dose of cinacalcet was given while three patients received 60 mg. Five patients received cinacalcet (30–120 mg) prior to the hospital admission, three patients with Ca in the 3.4–3.6 mmol/L range – as initial hypocalcemic therapy combined with isotonic saline right after admission. However, no significant decrease in Ca levels due to cinacalcet therapy was observed. In two patients, denosumab was administered concurrently with cinacalcet and saline hydration because of the severity of hypercalcemia (Ca baseline 3.94 and 4.42, respectively) and related symptoms. One female patient (No 3) initially received treatment with 90 mg of cinacalcet; the lack of effect required the addition of denosumab. Normocalcemia was achieved on 3rd day after injection and cinacalcet was canceled. In another patient (No 8) with parathyroid cancer and impaired renal function (eGFR 13 mL/min/1.73 m^2^) denosumab 60 mg was combined with cinacalcet 120 mg. We registered complete Ca normalization on the 10th day, then cinacalcet was gradually discontinued.

### Reduction of hypercalcemia and hypercalcemic-related symptoms

**Figure 1:**
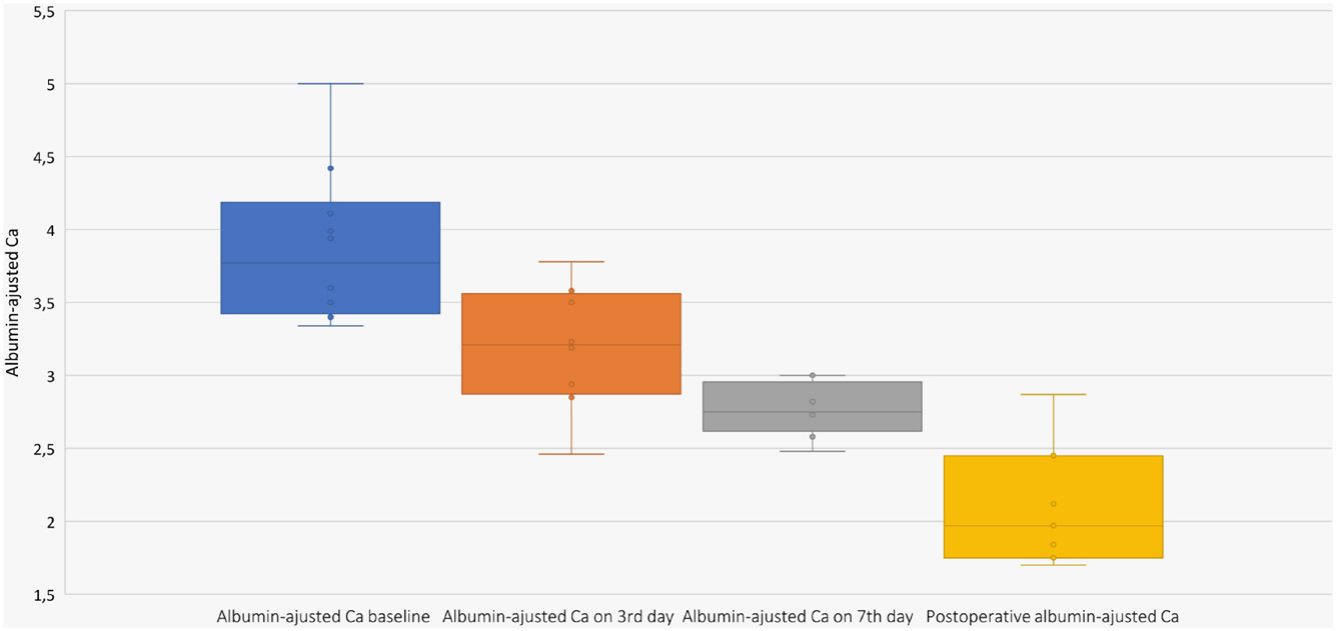
Follow-up changes of serum calcium level before and after denosumab injection (Friedman criterion, *P* = 0.002).

The trend to calcium reduction was already determined on the 3rd day after denosumab administration (Ca baseline vs 3rd day, *P* = 0.012). In most cases (8/10) the decrease in serum calcium level to the range of 2.8 mmol/L on average or lower was observed on the 7th day (range 3–9 days). Ca levels decreased progressively (comparing Ca before denosumab administration and on the 3rd and 7th day after injection, *P* = 0.002 (Fig. 1)). Normocalcemia was achieved in four patients: one on the 3rd day and the others on the 9th day. After stabilization of Сa levels, eight patients underwent a gradual withdrawal of cinacalcet. We did not notice the reverse effect in calcium concentrations after cinacalcet cancellation before surgery. In two patients, calcium levels were stabilized at nearly 3 mmol/L. As a result, cinacalcet was prolonged until surgical treatment.

Serum calcium reduction was accompanied by the improvement of patient’s clinical state. The earliest change was an decrease of dehydration that occurred in the first three days in most cases. By the 7th and 9th day, the elimination or reduction of neurological and gastroenterological symptoms, such as headaches, weakness, nausea, vomiting, were noticed in the majority of patients. The two patients (Nos 2 and 10) who previously had the highest calcium levels had little changes in dyspeptic disorders during that period.

### Changes in PTH

Data on dynamics of PTH levels after denosumab injection (before surgery) were available in seven patients. No significant changes were observed in four of them, a decrease was recorded in two patients, and only one patient with generalized *osteitis fibrosa cystica* showed an increase in PTH from 1871 to 5000 pg/mL on the 10th day after denosumab administration (Table 1), and stabilization of blood calcium level at 3 mmol/L. On the 13th day this patient was radically operated with the development of hypocalcemia from ‘hungry bone syndrome’.

### Increase in eGFR

**Figure 2:**
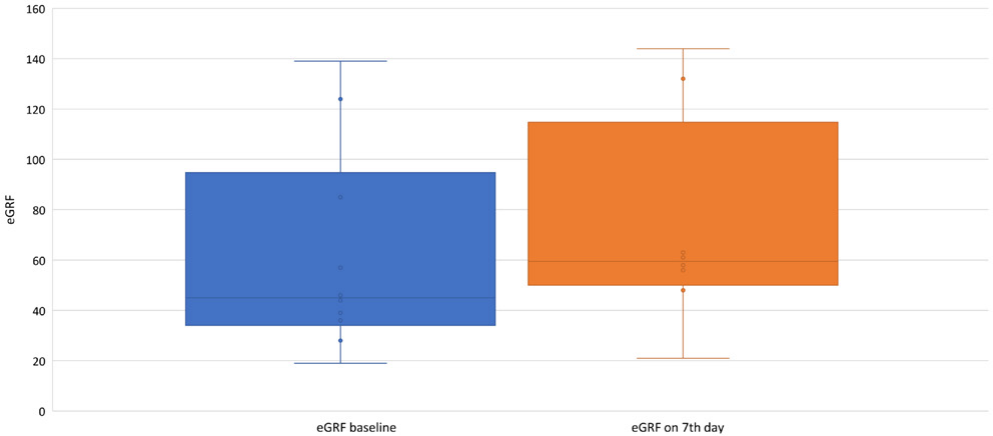
Follow-up changes of eGFR before and after denosumab injection (Wilcoxon criterion, *P* = 0.012).

A significant increase in eGFR was recorded on the 7th day after denosumab injection (eGFR baseline vs 7th day, *P* = 0.012). The dynamic of eGFR before and after injection is shown in the Fig. 2.

### Surgical treatment and follow-up

Seven patients underwent successful parathyroidectomy and achieved eucalcemia (*n* = 1) or hypocalcemia (*n* = 6, 1.97 mmol/L (1.7; 2.12)), which was successfully treated with *per os* active vitamin D and calcium supplements, not requiring calcium infusions. Pathology reports have not noted any bleeding or necrosis of parathyroid grands.

Two elderly patients with severe concomitant cardiovascular pathology categorically refused surgery. Biochemical monitoring in a month showed an increase in calcium level up to 3.2 mmol/L in one patient (No 1), which required the resumption of cinacalcet 60 mg (the patient continues to refuse surgery). During the next 2 months, Сa stabilized at 2.8–2.85 mmol/L. In second case (P No 2) the lab tests showed stable mild hypercalcemia 2.55–2.68 mmol/L with a follow-up period of 3 months. Interestingly, that low-normal Ca level was observed on the fourth month of therapy, 5 months after denosumab injection we detected an increase in the Ca level to and 2.32 mmol/L. One patient (No 9) developed the recurrence of parathyroid cancer. Because of unidentified metastases re-operation has been delayed. Denosumab was prescribed to control hypercalcemia. Pending results, the calcium level remains stable 1 month after the injection at 2.7–2.75 mmol/L.

## Discussion

Management of hypercalcemia should be aimed at both lowering the serum calcium and the elimination pathogenic factor, if possible. Effective therapy reduces serum calcium by inhibiting bone resorption, enhancing renal excretion or decreasing intestinal absorption of calcium. The optimal choice depends on the cause and severity of hypercalcemia. Mild and moderate forms of hypercalcemia are often well tolerated (11), while severe hypercalcemia is a life-threatening condition (3, 4). One of the most common causes of hypercalcemia is PHPT.

A retrospective review of 177 patients (12) who underwent surgical treatment of PHPT at a single institution, revealed 37 patients (21%) with severe hypercalcemia ≥3.5 mmol/L. The most frequent of presented symptoms were bone pain and fractures (81%), proximal muscles weakness (81%), and fatigue (83%). Mental status changes, kidney stones and nephrocalcinosis occurred less often – in 29, 24 and 19% of patients, respectively. Acute pancreatitis was found in 13%. This is totally consistent with our data as skeletal complications were identified most frequently, but as we noticed, the neurological disorders were more prevalent (60%).

Generally, the urgent management of hypercalcemia is based on intravenous rehydration with isotonic saline, which are usually combined with administration of loop diuretics, calcitonin, bisphosphonates or calcimimetics (6, 11). Dialysis is also an option for patients with severe hypercalcemia and renal insufficiency or heart failure, whom hydration cannot be safely administered (13).

The bisphosphonates show good evidence for protecting bone mineral density but no evidence for fracture risk reduction in PHPT and no consistent evidence for reduction of hypercalcaemia in PHPT or HC (4, 12, 14). However, therapy with bisphosphonates has some limitations and side effects including fever, which may exacerbate dehydration, bone pain during and post infusion, osteonecrosis of the jaw, uveitis, orbital inflammation, and possesses a risk of significant nephrotoxicity. Bisphosphonates may lead to decrease in eGFR and to permanent requirement in hemodialysis (4).

A calcium-lowering effect can be produced by cinacalcet, a calcimimetic that interacts with the calcium sensing receptor (СaSR) on parathyroid cells leading to the downregulation of PTH with an attendant decline in serum calcium levels (4). In our study all patients received cinacalcet in various doses for hypercalcemia management. Nevertheless, in eight patients cinacalcet was initially given before the denosumab injection and no significant calcium decrease was observed (median Ca 3.55 mmol/L). This can be probably explained by a delayed hypocalcemic effect of the drug, inability to properly escalate the dose due to drug intolerance, or lack of CaSR sensitivity at the tumor level. The efficacy of cinacalcet in the management of parathyroid cancer-related hypercalcemia was initially assessed in an open-label single-arm study of 29 patients (dose titration from 60 to 360 mg daily). Over the 16-week study period, 62% of patients responded to therapy – serum calcium was reduced by at least 0.25 mmol/L; the greatest response was seen in patients with the highest baseline calcium levels. A decrease in PTH levels was not clinically significant (7). So, the clinically significant effect of cinacalcet can be achieved only at high doses, which are very hard to attain.

More recently, the novel inhibitor of osteoclast function denosumab has been shown to have a useful role as a hypocalcemic agent. Denosumab could be a treatment of first choice for PTH-related hypercalcemia, especially in severe renal impairment or when bisphosphonates are ineffective. Evidence suggests that denosumab could be effective in increasing BMD and lowering increased by PTH bone turnover in patients with PHPT (10, 15). According to DENOCINA study of PHPT patients (key inclusion criteria were a T-score between −1.0 and −3.5 at the lumbar spine, femoral neck, or total hip) (10), plasma calcium decreased significantly in the denosumab group during the first month, but afterwards it returned to baseline concentrations (all patients had baseline mild hypercalcemia). Normocalcemia was achieved only in combined group denosumab+cinaclcet (in 64% of patients).

The data on denosumab use in patients with severe resistant hypercalcemia secondary to PHPT is limited. There is description of individual clinical cases, more often of hypercalcemia related to parathyroid carcinoma. Hsu *et al.* described a 74-year-old woman with metastatic parathyroid carcinoma, managed with multiple medications including bisphosphonates, calcitonin or cinacalcet and even palliative radiotherapy with substandard effect (16). Only the use of 60 mg of denosumab allowed to normalize of calcium levels and the result of this achievement continued for several months (16). Von Schwartzenberg *et al.* reported a calcium-lowering effect of denosumab for uncontrollable hypercalcemia due to parathyrotoxicosis which was achieved after 60 mg subcutaneous injection in 1 day. Calcium levels remained stable between 2.6 and 2.7 mmol/L for 4 months, after another 60 mg of denosumab was injected. The long-term follow-up for more than 2 years showed that repeated low-dose denosumab injections (a summary six doses) were effective in maintaining stable calcium levels despite rising PTH and ineffective surgeries (17). Similar to this observation, calcium levels dropped rapidly within 2 days after denosumab injections and remained stable for about a month in a patient with renal cell carcinoma, lung metastases and bisphosphonate-refractory hypercalcemia (18). Remya Rajan *et al.* reported a case of PHPT with severe hypercalcemia (3.7 mmol/L) against the background of renal dysfunction (chronic kidney disease and with medullary nephrocalcinosis with eGFR of 21.35 mL/min/1.73 m^2^), in whom denosumab (60 mg subcutaneously) was used to lower the calcium levels prior to surgery. Serum calcium decreased on the 3rd day. After curative parathyroid surgery, calcium remained normal and there was no evidence of ‘hungry bone’ syndrome (19).

The question of the onset and completion of denosumab with regard to hypocalcemic effect in PHPT remains open. The median time to response (time taken to lower calcium <2.87 mmol/L) for denosumab in the management of malignant hypercalcemia has been described as 9 days (range 7–10) (9). The international study of Hu *et al.* showed the calcium-lowering effect of denosumab in 33 patients with malignant hypercalcemia in 10 days. The median response duration to denosumab (time from initial response to last day serum calcium ≤2.9 mmol/L) was 104 days (20). These results are in line with our findings as the significant decrease in serum calcium level to the range of 2.8 mmol/L or lower on average was observed on the 7th day (*P* = 0.002), however, in one patient, calcium normalization was observed as early as on the 3rd day after injection. Serum calcium level stabilized at 3 mmol/L in two patients. The absence of the desired effect in these cases can presumably be explained by the levels of PTH and AP (1871 and 1423 pg/mL; 461 and 502 U/L, respectively) and the presence of generalized *osteitis fibrosa cystica*, confirming extremely high bone resorption.

The effect of denosumab on PTH levels in our study was controversial. The main limitation is that lab tests were performed on different days after the drug administration. In most patients, PTH levels remained stable before surgery or even slightly decreased. Given the good hypocalcemic effect of the drug, it is difficult to explain these changes. In the DENOCINA trial plasma PTH increased rapidly after denosumab administration and then slowly, but significantly declined. Several previous studies on denosumab in postmenopausal osteoporosis have also shown the temporary increases in plasma PTH after the injection.

Denosumab can lead to hypocalcemia, especially in patients severe renal impairment (eGFR <30 mL/min/1.73 m^2^). It is also associated with osteonecrosis of the jaw similar to parenteral bisphosphonates but this side effect is extremely rare and is usually associated with profound and long inhibition of bone resorption, and is not seen from a single injection. Besides, it does not have nephrotoxic effect and dose adjustment is not required depending on renal function. The improvement in the filtration function in our study may be explained both through active rehydration and a significant reduction in calcium levels. Severe hypercalcemia may lead to acute kidney injury by direct renal vasoconstriction and by decreases in extracellular fluid volume (21).

Most of our patients underwent successful parathyroidectomy within several weeks of their injection; postoperative hypocalcemia was corrected with active vitamin D and calcium supplements *per os*. It is not possible to estimate how much denosumab influenced the course of postoperative hypocalcemia. However, the main reasons for its development include transient hypoparathyroidism and ‘hungry bone’ syndrome in patients with severe skeletal impairment.

Longer follow-up assessment of the denosumab effect was possible in three cases. With a follow-up period of maximum 5 months, in two patients calcium levels remained stable (lower than 2.8 mmol/L), while one patient had a relapse of severe hypercalcemia, which required the resumption of cinacalcet. In recently reported cases of parathyroid-cancer related to hypercalcemia and also related to parathyroid carcinoma, dosing intervals of denosumab to achieve target calcium levels were as short as 1 month (2, 22, 23). However, there is evidence that hypercalcemia can be controlled by less frequent injection, up to 3–4 months without adverse events (17, 24).

### Limitations

The large heterogeneity of the study population with different types of PTH-related hypercalcemia (carcinoma and adenoma of parathyroid glands), different treatment schedules for the isotonic saline infusion and cinacalcet does not allow to fully estimate the impact of denosumab on serum calcium.

## Conclusion

The effect of denosumab on RANKL signaling, inhibition of PTH-driven bone resorption and associated reduction of serum calcium levels makes it a useful tool to control severe hypercalcemia in patients with PHPT before surgery or if surgery is contraindicated for some reasons. Benefits include no restrictions for denosumab use in patients with concomitant chronic kidney disease.

## Declaration of interest

The authors declare that there is no conflict of interest that could be perceived as prejudicing the impartiality of the research reported.

## Funding

This research was conducted using funds of the state assignment # AAAA-A18-118051590060-2.
